# Supplementary material for: Dynalign II: common secondary structure prediction for RNA homologs with domain insertions
Source: Nucleic Acids Res. 2014 Nov 21;42(22):13939–48. doi: 10.1093/nar/gku1172 (PMC4267632; doi:10.1093/nar/gku1172)
Supplement: SUPPLEMENTARY DATA [file supp_gku1172_nar-02021-z-2014-File012.zip › manual/Text/EnsembleEnergy.html]

RNAstructure Command Line Help: EnsembleEnergy


|  |  |  |
| --- | --- | --- |
|  | RNAstructure Command Line Help EnsembleEnergy | - Contents - Index |
| EnsembleEnergy calculates the energy of a structure ensemble from either a partition function save file or a sequence file. This ensemble energy is -RT ln(Q), where Q is the partition function. It prints results on standard output, so no output file is generated. USAGE: EnsembleEnergy <input file> [options] Required parameters: <input file> \| The name of a file containing input data. This input data can be in one of two formats: 1. Partition function save file (holds base pairing probability data for all pairs and can be generated using the partition interface). 2. Sequence file (holds raw sequence: .seq or .fasta). Note that lowercase nucleotides are forced single-stranded in structure prediction. Note that in order to use a squence file, the "sequence" flag must be specified (see "--sequence" below). Options which don't require added values: -d, -D, --DNA \| This flag only matters if the input file is a squence file and has been identified as such (see "--sequence" below). Specify that the sequence is DNA, and DNA parameters are to be used. Default is to use RNA parameters.; -h, -H, --help \| Display the usage details message.; -s, -S, --silent \| Suppress all progress messages except the final ensemble energy result. Note that this does NOT suppress errors, so errors will be shown even if this option is specified.; --sequence \| Identify the input file format as a sequence file. Options which require added values: NONE References: 1. Reuter, J.S. and Mathews, D.H. "RNAstructure: software for RNA secondary structure prediction and analysis." *BMC Bioinformatics*, 11:129. (2010). | | |
| Visit The Mathews Lab RNAstructure Page for updates and latest information. | | |
